# Human herpesvirus 6B encephalitis in a liver transplant recipient: A case report and review of the literature

**DOI:** 10.1111/tid.13403

**Published:** 2020-07-21

**Authors:** Yinfeng Wang, Di Wang, Xiaogen Tao

**Affiliations:** ^1^ Department of Intensive Care Medicine The First Affiliated Hospital of USTC Division of Life Sciences and Medicine University of Science and Technology of China Hefei Anhui 230036 China

**Keywords:** HHV‐6B infection, liver transplantation, next‐generation sequencing, viral encephalitis

## Abstract

Human herpesvirus 6B (HHV‐6B) encephalitis in a liver transplant recipient is rarely reported. In this report, we presented a case of HHV‐6B encephalitis in a liver transplant recipient and reviewed the relevant literature. A 56‐year‐old man was admitted to the intensive care unit (ICU) with an acute headache and intermittent convulsion 17 days after liver transplantation. Next‐generation sequencing (NGS) of the cerebrospinal fluid (CSF) revealed 30691 sequence reads of HHV‐6B and real‐time polymerase chain reaction (real‐time PCR) of the CSF detected HHV‐6B DNA at 12 000 copies/mL, so the patient was diagnosed with HHV‐6B encephalitis and received ganciclovir treatment promptly. The condition of the patient improved well and returned to the general ward with no neurologic deficits. This case indicated that adequate awareness, early diagnosis, and timely treatment are crucial to a good prognosis of HHV‐6B encephalitis after liver transplantation.

## 1 INTRODUCTION

Human herpesvirus 6 (HHV‐6) is a β‐herpesvirus and consists of two genetically distinct variants: HHV‐6A and HHV‐6B. Most HHV‐6 infection is asymptomatic or manifests as diarrhea, non­specific fever, and rash. However, HHV‐6–induced encephalitis can occur after transplantation and is associated with poor prognosis.^1^ HHV‐6 encephalitis after liver transplantation has rarely been reported. Here, we present a case of human herpesvirus 6B encephalitis in a liver transplant recipient and review the relevant literature.

## 2 MEDICAL HISTORY

A 56‐year‐old man with liver tumor recurrence after curative surgery was admitted to the hospital and received orthotopic liver transplantation. He received the medication regimen of tacrolimus and sirolimus for immunosuppressive therapy post‐surgery. This patient developed a headache, intermittent convulsion, and the disturbance of consciousness 17 days after transplantation and then was transferred to our department.

## 3 PHYSICAL EXAMINATION

The temperature was 37.2°C, pulse was 147 beats/min, and blood pressure was 137/91 mm Hg, SpO2 of 88%‐92% (oxygen concentration: 5 L/min), double pupils of equal size and equal circle, the diameter of about 2.5 mm, sensitive to light reflection, and the appearance of an erythematous cutaneous rash on the trunk and back. The patient was unable to follow commands, physical examination revealed no evidence of Babinski's sign, and muscle strength and muscle tone were normal.

## 4 LABORATORY EXAMINATIONS

Additional laboratory values included the following: C‐reactive protein 7 mg/L, ALT 46 IU/L, AST 30 IU/L, 46 μmol/L, blood ammonia 46 μmol/L, and BNP <100 pg/mL. Imaging of the brain by CT scanning and magnetic resonance imaging (MRI) demonstrated no other abnormalities except for cerebral ischemia, and the electroencephalogram was abnormal due to the continuous electrographic seizures. Cerebrospinal fluid (CSF) pressure was 240 mH_2_O, and the CSF test indicated the following: nucleated cell counts 20 × 10^6^/L, red blood cells 0, protein 0.1 g/L, glucose 4 mmol/L, and chloride 119.9 mmol/L.

## 5 TREATMENT AND DIAGNOSIS

**Figure 1:**
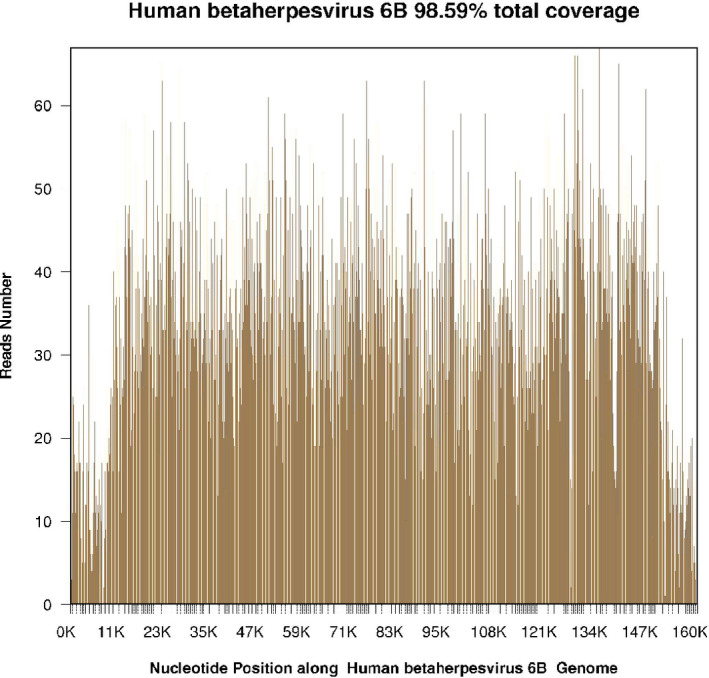
Next‐generation sequencing (NGS) of cerebrospinal fluid (CSF) showed 30691 sequence reads of HHV‐6B with the coverage of 98.59% on its genome

**Figure 2:**
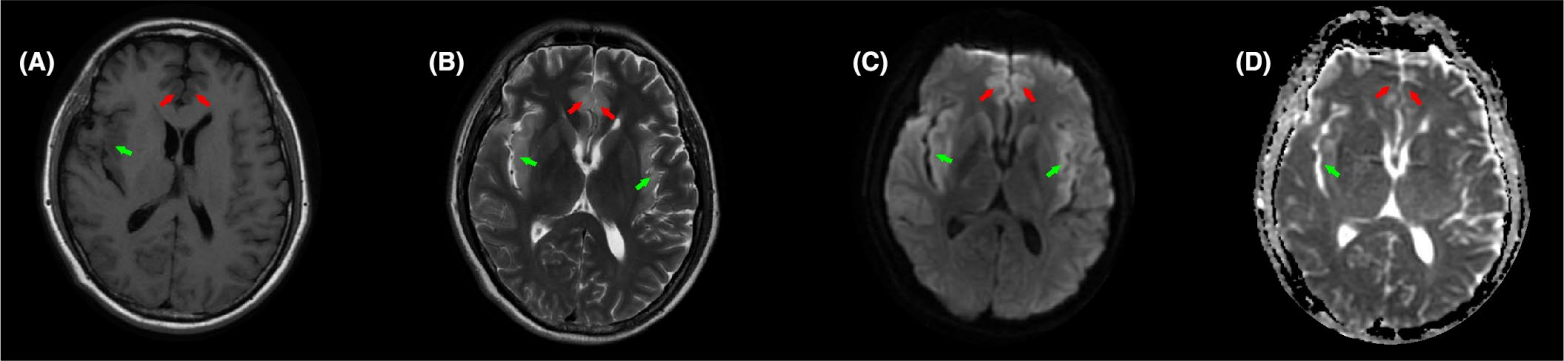
Cranial MRI showed hyperintense signal distributed symmetrically in bilateral frontal lobe (A and B, red arrow) and temporal lobe (A and B, green arrow). Diffusion‐weighted MRI showed hyperintense signal distributed symmetrically in bilateral frontal lobe (C, red arrow) and temporal lobe (C, green arrow). Apparent diffusion coefficient MRI showed hypointense signal distributed in bilateral frontal lobe (D, red arrow), and right temporal lobe (D, green arrow)

**Figure 3:**
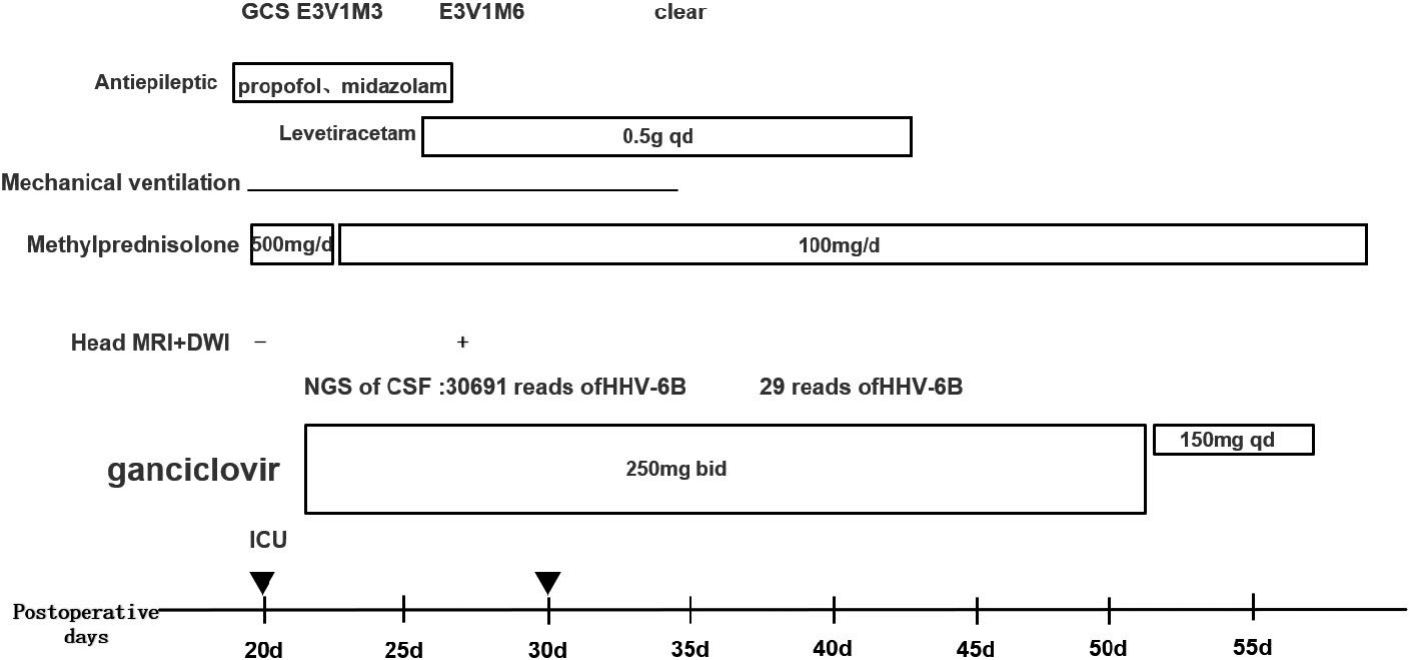
Clinical course of Human herpesvirus 6B (HHV‐6B) encephalitis in a liver transplant recipient

On admission, the patient was diagnosed with epilepsy of unknown cause and was treated with mechanical ventilatory support and antiepileptic drugs. Then, tacrolimus and sirolimus were discontinued, and glucocorticoid was initiated. Various B vitamins were supplemented to improve neurologic symptoms. Simultaneously, a next‐generation sequencing assay was performed to identify central nervous system infection. Two days later, next‐generation sequencing (NGS) of CSF showed 30691 sequence reads of HHV‐6B with 98.59% coverage of the HHV‐6B genome (Figure 1) and real‐time polymerase chain reaction (real‐time PCR) of the CSF detected HHV‐6B DNA at 12 000 copies/mL, so the patient was diagnosed with HHV‐6B encephalitis and received intravenous ganciclovir (250 mg daily) immediately. MRI scan of the brain showed symmetric hyperintense signal distributed in the frontal lobe and temporal lobe on the fourth day of ICU admission (Figure 2). 2 weeks after starting ganciclovir, the patient's clinical manifestations markedly improved with the stable neurologic status, and real‐time PCR of HHV‐6B were all negative (blow 1000 copies/mL) for CSF and blood. The patient was weaned from the ventilator on the next day. After ganciclovir administration for four weeks, CSF pressure returned to 80 mmH_2_O. From this day onwards, the dosage of ganciclovir decreased to 150 mg/d. Thirty‐seven days after admission, he was discharged to the organ transplant ward for further treatment, with no obvious neurologic deficits. The clinical course was summarized in Figure 3.

## 6 DISCUSSION

Human herpesvirus 6 is a widespread virus that primarily infects most children in the first 2 years of age.^2^ Seroprevalence of HHV‐6 in healthy adulthood is up to 90%, so most HHV‐6 infections in transplantation recipients are thought to be caused by virus reactivation. The prevalence of HHV‐6 reactivation among liver transplantation recipients varies widely from 4.3% to 53.7%, partly due to differences in study size and diagnostic assays for HHV‐6.^3^

Most HHV‐6 reactivations in liver transplantation recipients are transient and asymptomatic with a low level of HHV‐6 DNA.^4^ However, three cases of severe HHV‐6 encephalitis after liver transplantation have been reported.^5^, ^6^, ^7^ All three patients initially present with confusion, among which one patient also developed generalized tonic‐clonic seizures. Cranial MRI of three patients was characterized by symmetric high signal intensity predominantly in the medial temporal lobes, which contained the hippocampi and amygdala. Of all three patients, PCR results of CSF were positive for HHV‐6, leading to the diagnosis of HHV‐6 encephalitis. Ganciclovir and phosphonoformic acid (foscarnet) are currently recommended first‐line treatments for HHV‐6B.^8^ Among three patients, one patient received intravenous ganciclovir, and the other two patients received intravenous phosphonoformic acid, respectively, due to ganciclovir‐resistance and ganciclovir‐induced leukopenia. All three patients recovered well without detectable neurologic deficits. The patient in our report started with seizures and impaired consciousness, and cranial MRI revealed symmetric high signal intensity in the frontal lobe and medial temporal lobes. HHV‐6 was positive in CSF with a high DNA level. After four weeks of treatment of ganciclovir, the patient's mental functioning had recovered to his baseline. One‐third of HHV‐6B encephalitis in allogeneic hematopoietic transplantation recipients have been reported to be specifically diagnosed with post‐transplant limbic encephalopathy, which is characterized with temporal seizures, anterograde amnesia, syndrome of inappropriate antidiuretic hormone secretion, and the abnormal signal in the limbic system by MRI.^9^ From the current four reported cases, the clinical characteristics of HHV‐6 encephalitis in liver transplantation recipients are mostly consistent with those of post‐transplant limbic encephalopathy. However, more cases are needed for further investigation.

The indirect adverse effect of HHV‐6 infection on liver transplantation has been studied. HHV‐6 positivity in blood or bile was associated with the incidence of liver allograft rejection.^10^, ^11^, ^12^ In liver transplantation recipients, HHV‐6 infection also results in increased risks of severe opportunistic infections, including CMV, EBV, VZV, mycobacterial disease, and invasive fungal infections.^3^, ^13^ NGS, known as high‐throughput sequencing, can detect unexpected pathogens in a single application. NGS has been proved to be a promising method to identify abundant and transplant‐related pathogens.^14^ In this case, NGS found sequence reads of HHV‐6B in the patient's CSF, which facilitated the timely diagnosis of HHV‐6B encephalitis and contributed to a favorable prognosis of the patient.

Human herpesvirus 6 has a unique ability to integrate its genome into telomeres of the human host. This phenomenon, commonly called inherited chromosomally integrated HHV‐6 (ciHHV‐6), occurs in 2% of liver transplants patients.^3^ Recognition of ciHHV‐6 is vital for clinical strategy. ciHHV‐6–induced HHV‐6 infection after liver transplant has been reported to be associated with liver rejection and mortality.^15^, ^16^ On the other hand, misdiagnosis of ciHHV‐6 as HHV‐6 reactivation will result in unnecessary and potentially toxic antiviral therapy.^17^ Thus, it has been recommended to examine donated organs and organ recipients for ciHHV‐6. If HHV‐6 load in whole blood is above 5.5 log10 copies/mL or persistently at a high level under appropriate antiviral treatment, ciHHV‐6 should be assumed and further confirmed by quantitative PCR of HHV‐6 on fingernails or hair follicles.^18^ In this case, quantitative PCR of HHV‐6 revealed 12 000 copies/mL (4.08 log10 copies/mL) in CSF and negative results in whole blood, which excluded the possibility of ciHHV‐6 and indicated that the encephalitis might be caused by endogenous reactivation of HHV‐6B strain. However, we did not determine ciHHV‐6 status in the donor, which is the inadequacy of this case report.

## 7 CONCLUSION

In summary, HHV‐6 infection should be considered if liver transplant recipients develop confusion or epileptic seizures. The prompt early diagnosis and the timely application of antiviral therapy are of great significance for improving the patient's prognosis.

## CONFLICT OF INTEREST

There are no conflicts of interest.

## AUTHOR CONTRIBUTIONS

Yinfeng Wang and Di Wang wrote the manuscript, and Xiaogen Tao revised it.
